# A Subregion of Reelin Suppresses Lipoprotein-Induced Cholesterol Accumulation in Macrophages

**DOI:** 10.1371/journal.pone.0136895

**Published:** 2015-08-28

**Authors:** Emmanuel U. Okoro, Hongfeng Zhang, Zhongmao Guo, Fang Yang, Carlie Smith, Hong Yang

**Affiliations:** 1 Department of Physiology, Meharry Medical College, Nashville, Tennessee, United States of America; 2 Department of Pathology, Central Hospital of Wuhan, Wuhan City, People’s Republic of China; 3 Wuhan University School of Basic Medical Science, Wuhan City, People’s Republic of China; Pennsylvania State Hershey College of Medicine, UNITED STATES

## Abstract

Activation of apolipoprotein E receptor-2 (apoER2) and very low density lipoprotein receptor (VLDLR) inhibits foam cell formation. Reelin is a ligand of these receptors. Here we generated two reelin subregions containing the receptor binding domain with or without its C-terminal region (R5-6C and R5-6, respectively) and studied the impact of these peptides on macrophage cholesterol metabolism. We found that both R5-6C and R5-6 can be secreted by cells. Purified R5-6 protein can bind apoER2 and VLDLR. Overexpression of apoER2 in macrophages increased the amount of R5-6 bound to the cell surface. Treatment of macrophages with 0.2 μg/ml R5-6 elevated ATP binding cassette A1 (ABCA1) protein level by ~72% and apoAI-mediated cholesterol efflux by ~39%. In addition, the medium harvested from cells overexpressing R5-6 or R5-6C (R5-6- and R5-6C-conditioned media, respectively) also up-regulated ABCA1 protein expression, which was associated with accelerated cholesterol efflux and enhanced phosphorylation of phosphatidylinositol 3 kinase (PI3K) and specificity protein-1 (Sp1) in macrophages. The increased ABCA1 expression and cholesterol efflux by R5-6- and R5-6C-conditioned media were diminished by Sp1 or PI3K inhibitors mithramycin A and LY294002. Further, the cholesterol accumulation induced by apoB-containing, apoE-free lipoproteins was significantly less in macrophages incubated with R5-6- or R5-6C-conditioned medium than in those incubated with control conditioned medium. Knockdown of apoER2 or VLDLR attenuated the inhibitory role of R5-6-conditioned medium against lipoprotein-induced cholesterol accumulation. These results suggest that the reelin subregion R5-6 can serve as a tool for studying the role of apoER2 and VLDLR in atherogenesis.

## Introduction

Apolipoprotein E receptor-2 (apoER2) and very low density lipoprotein receptor (VLDLR) belong to the low-density lipoprotein receptor family. Since these receptors are highly expressed in endothelial cells, smooth muscle cells and macrophages ^[^ ^1^ ^–^ ^5^ ^]^, there is significant interest in studying their involvement in atherosclerosis. However, data from previous studies are inconsistent. Specifically, it has been reported that transplantation of VLDLR-expressing macrophages into VLDLR-deficient mice accelerated atherosclerosis development [6]. In contrast, VLDLR deficiency increased intimal thickening after vascular injury and increased necrosis in atherosclerotic lesions [1]. Deficiency in apoER2 also enhanced macrophage susceptibility to oxidized low density lipoprotein (oxLDL)-mediated lipid accumulation and cell death, and augmented atherosclerotic plaque progression and necrosis [7]. Similarly, studies using cultured cells showed that activation of VLDLR and apoER2 can induce either pro- or anti-atherogenic effects. For instance, activation of VLDLR and/or apoER2 by native and oxidized apoB-containing lipoproteins [8,9], antiphospholipid antibodies [5], human neutrophil peptides [4], and coagulation factor XI [10] elevated intracellular cholesterol accumulation and induced cell adhesion, possibly by activation of a p38-mediated pathway [8,9]. In contrast, activation of these receptors by activated protein C (APC) [11] and apoE [12] have been shown to inhibit cellular events that potentially contribute to inflammation. Recently, we reported that activation of apoER2 and VLDLR by apoE increased ATP-binding cassette transporter A1 (ABCA1) expression and cholesterol efflux by triggering a signaling cascade including disabled-1 (Dab1), phosphatidylinositol 3-kinase (PI3K), protein kinase C-ζ (PKC-ζ) and specificity protein 1 (Sp1) [13–15]. Taken together, it appears that the effect of apoER2 and VLDLR on atherogenesis depends on the interacting ligands and the signaling pathways involved.

Reelin is a natural ligand of apoER2 and VLDLR, and is highly expressed in the brain. Interaction of reelin with apoER2 and VLDLR regulates neuronal cell migration and positioning during development and modulates synaptic plasticity in the adult brain [16]. Deficiency of reelin has been implicated in a number of neurological disorders, such as lissencephaly and Alzheimer’s disease. Reelin is also expressed in lymphatic endothelial cells, where it regulates lymphatic vessel development and function [17]. Far less is known about its role, if any, in other peripheral tissues, even though it is detectable in some peripheral tissues, such as the liver and blood [18]. Reelin consists of 3461 amino acids and is organized into a multi-domain N-terminal region, eight tandem repeats and a well-conserved C-terminal region (CTR). It is cleaved in vivo at two sites located between repeats 2–3 and between repeats 6–7, resulting in the production of 3 fragments [19]. It has been suggested that the central fragment, which consists of repeats 3–6, is sufficient for reelin functions. The receptor binding domain is located in repeats 5 and 6 [19,20]. However, the reelin subregion containing only repeats 5 and 6 reportedly enables [20] or fails [19] to induce cellular responses as the reelin central fragment. Previous studies also provide conflicting evidence regarding the function of the reelin CTR, *i*.*e*., this arginine-rich region is reported to be either essential [21] or dispensable [22] for reelin secretion.

In this report, we generated two reelin subregion polypeptides, one contains the 5^th^ and 6^th^ repeats of reelin (designated as R5-6), while another contains R5-6 and the CTR (designated as R5-6C). We observed that both R5-6 and R5-6C can be secreted from cells. These suggest that the reelin CTR is not required for cells to secrete R5-6. Our data also suggest that R5-6 is able to upregulate ABCA1 expression, enhance cholesterol efflux, and diminish lipoprotein-induced cholesterol accumulation in macrophages by activation of a signaling pathway involving apoER2/VLDLR, PI3K, and Sp1.

## Materials and Methods

### Ethics Statement

All procedures for handling animals were conducted following protocols approved by the Institutional Animal Care and Use Committee at Meharry Medical College, protocol number 080820HY161, entitled “Defective Isoforms of ApoE-Induced Atherogenesis via Unfolded Protein Responses.”

### Cells and Reagents

RAW 264.7 macrophages (TIB-71) and HEK 293 (CRL-1573) cells were obtained from ATCC (Manassas, VA). PI3K inhibitor LY294002 (sc-201426), Sp1 inhibitor mithramycin A (sc-200909), ELISA plate (sc-204463), scrambled siRNA, and siRNAs specific for VLDLR or apoER2, antibodies against ABCA1 (sc-58219), β-actin (sc-47778), apoER2 (sc-20746), VLDLR (sc-18824), and mouse IgG were purchased from Santa Cruz Biotechnology (Santa Cruz, CA). Dulbeco’s Modified Eagle’s Medium (DMEM), culture medium OptiMEM, c-myc antibody (R950-25), anti-c-myc agarose beads (cat. # 20168), pcDNA3.1B plasmid, EcoRV, HindIII and XhoI were purchased from Life Technologies (Carlbad, CA). Mouse apoER2-GFP expression plasmid (MG226275) was purchased from Origene (Rockville, MD). Antibodies against PI3K (#4249) and phosphorylated PI3K (p-PI3K) (#4228) were obtained from Cell Signaling (Beverly, MA), while antibodies against apoER2 (ab58216) and VLDLR (ab92943) were obtained from Abcam (Cambridge, MA). Phospho-Sp1 antibody (A7218) was purchased from Assay Biotechnology (Sunnyvale, CA). The pfu ultra DNA polymerase was purchased from Agilent Technologies (Santa Clara, CA). [1,2-^3^H(N)]-cholesterol was obtained from American Radiolabeled Chemicals (St. Louis, MO). Cholesterol assay reagents were obtained from Wako Diagnostics (Richmond, VA). Fetal bovine serum (FBS) was obtained from Atlanta Biologicals (Flowery Branch, GA). Amicon Ultra centrifugal filter (cat. # UFC501024) was purchased from Millipore (Darmstadt, Germany). The 1-StepUltra TMB-ELISA Substrate Solution (cat. # 34028) was obtained from Thermo Scientific (Rockford, IL).

### Recombinant Plasmid Construction and Cell Transfection

The pCrl plasmid containing the full-length reelin central fragment cDNA was a gift from Gabriella D’Arcangelo and Tom Curran (Rutgers University, Piscataway, NJ). Two fragments were amplified by PCR from this pCrl plasmid using pfu ultra DNA polymerase. One of these fragments corresponds to amino acids 1918–2664, which include the sequence of the 5^th^ and 6^th^ repeats of reelin. Another fragment corresponds to amino acids 3365–3461, which include the 32 amino acids of the reelin CTR (3430–3461). The following primers were used for generation of these DNA fragments: R5-6 forward: 5’-atcccaagcttatggcccaaaccaacgctacaag-3’, R5-6 reverse: 5’-atccggatatcagagcccgagatgaggacat-3’; CTR forward: 5’-atccggatatcagtgtcaacaatggcatcac-3’, and CTR reverse: 5’-atccgctcgagcgtgggtatcgcctaagcgacc-3’. The PCR products of R5-6 were cloned into the HindIII/EcoRV sites of pcDNA3.1B, while the CTR was ligated with R5-6 in pCDNA3.1B using EcoRV/XhoI sites, in frame with c-myc-his epitopes. The final sequences were verified by DNA sequencing.

The recombinant plasmid constructs or the empty pcDNA3.1B vector was transfected into HEK 293 and RAW 264.7 cells by electroporation. Briefly, cells were equilibrated in pre-electroporation buffer consisting of phosphate buffered saline (PBS), OptiMEM, and FBS in a 1:8:1 ratio for 4 h. About 15 μg of the appropriate DNA in water was mixed with 9 volumes of electroporation buffer (pH 7.4): 50 mM KH_2_P0_4_, 10 mM HEPES, 250 mM sucrose, 10 mM EGTA, 10 mM MgCl_2_, and 10 mM glutathione. Electroporation was performed using a Nucleofect II device (Amaxa Biosystems, Gaithersburg, MD). The electroporated cells were mixed with 10 volumes of OptiMEM and incubated in 6-well plates at 37°C for 18 h. The media were removed of detached cells by centrifugation at 16,000 x g for 10 min at 4°C or filtration through a 0.25 μm Millipore filter. The supernatants/filtrates were analyzed by immunoblotting for detection of secreted proteins. Alternatively, supernatants/filtrates were mixed with 2/3 volume of fresh DMEM. The media derived from cells transfected with empty vector, R5-6 and R5-6C expression plasmids were designated as control, R5-6- and R5-6C-conditioned media, respectively. Fresh conditioned media were prepared for each experiment.

### Partial Purification of R5-6 and R5-6C

DH5α E.coli were transformed with the R5-6- or R5-6C-carrying pcDNA3.1B plasmid or empty pcDNA3.1B. The transformed bacteria was lysed at room temperature for 30 min with NTT lysis buffer (1% Triton X-100, 1% Tween-20, 150 mM NaCl, 40 mM Tris-HCl, pH 7.5) containing 1% of protease inhibitor cocktail and 1 mg/ml lysozyme. The lysate was centrifuged at 16000 x g for 10 min at 4°C. The supernatant was diluted 5 folds with 0.5% bovine serum albumin in TBS-T (150 mM NaCl, 20 mM Tris-HCl, pH 7.5, and 0.1% Tween-20) containing 0.1% sodium azide, 20 μM pepstatin A, 5 μM lactacystin, 50 μM ALLN, 1 mM leupeptin, and 10 μM aprotinin, and incubated with anti-c-myc agarose beads overnight at 4°C. After 3 washes with TBS-T and 2 with TBS (150 mM NaCl and 20 mM Tris-HCl, pH 7.5), R5-6 and R5-6C proteins associated with the agarose beads were eluted with c-myc peptide in TBS. The c-myc peptide in the eluent was removed by filtration using Amicon Ultra filter.

### Enzyme Linked Immunosorbent Assay (ELISA)

For cell surface ELISA [23], RAW 264.7 cells were electroporated with apoER2-GFP expression plasmid or pcDNA3.1B empty vector, followed by culturing in 96-well plates at 37°C for 24 h. The cells were then washed with PBS and fixed with 0.5% formaldehyde at 4°C for 30 min. It has been suggested that formaldehyde fixation does not significantly affect the binding activity of cell surface proteins [24]. After removing the fixative and washing twice with PBS, the fixed cells were incubated in 5% bovine serum albumin (BSA) ± 0.01 μg/ml R5-6 at 4°C for 8 h, with or without lipoprotein. The cells were then washed twice with TBS-T, and incubated with c-myc antibody or normal mouse IgG as a control for overnight. After two washes with TBS-T, the cells were incubated with horse radish peroxidase (HRP)-conjugated secondary antibody for 1 h in 5% BSA. Following one TBS-T wash, the ELISA was developed with TMB-ELISA substrate (Thermo Scientific). The absorbance was recorded at 450 nm using a microplate reader (Dynex Technologies).

For protein immobilization-based sandwich ELISA [25], the null eluent from empty-vector transformed bacteria, 1 μg/ml of c-myc peptide or 1 μg/ml of R5-6 or R5-6C in 0.2 M NaCO_3_-HCl (pH 9.6) was immobilized onto ELISA plates by incubation at room temperature for 5 h. The plates were washed with TBS and blocked with 5% fat-free milk. RAW 264.7 cells were lysed with NTT lysis buffer containing 1% of protease inhibitor cocktail and 1 mg/ml lysozyme. The lysate was centrifuged at 16000 x g for 10 min at 4°C, and the resulting supernatant was added to the protein-immobilized plates. Following overnight incubation at 4°C, the plate was washed once with TBS, and then incubated with antibodies against apoER2 or VLDLR in 5% fat-free milk at room temperature for 2 h. After one wash with TBS, the plate was sequentially incubated with HRP-conjugated secondary antibody and TMB-ELISA substrate. The developed color was read at 450 nm wavelength.

### Mouse Plasma Lipoprotein Isolation

Eight male apoE knockout (*apoE* ^*-/-*^ *)* mice (3–4 months old) were obtained from The Jackson Laboratory (Bar Harbor, ME), housed in groups of four for 5 days in Harlan GM500 cages (391x199x172 mm) bedded with soft cob bedding (Harlan Teklad, Madison, WI), and had free access to water and rodent chow diet (#5053, PicoLab, St. Louis, MO). Environmental conditions were a temperature of 21 ± 2°C, humidity of 55% ± 10%, and a 12:12 light:dark cycle with lights on at 0700 and off at 1900. At the start of the experiments animals weighed 19 ± 2 g. Approximately 0.5 ml of blood was collected from the posterior vena cava of mice under anesthesia with ketamine hydrochloride (100 mg/ml) at 0.8 μl/g body weight. Collected blood was immediately mixed with 50 μM butylated hydroxytoluene and 2 mM EDTA, and cooled on ice. Mouse plasma was adjusted to 1.063 g/ml and overlaid with buffered KBr (1.063 g/ml) and centrifuged at 125,749 x g for 18 h at 4°C, using a 70 Ti rotor (Beckman Coulter). Lipoproteins with density <1.063 g/ml were collected, dialyzed in PBS (pH 7.4) containing 1 mM EDTA at 4°C for 24–48 h, followed by dialysis in PBS without EDTA for 8 h and filtration through a 0.45-μm filter (44, 46). Aliquots of lipoproteins were stored at -80°C and utilized within 4 weeks. Storage of these lipoproteins at -80°C for 4 weeks did not significantly increase oxidation measured by thiobarbituric acid reactive substances (data not shown). These lipoproteins include VLDL, LDL, and chylomicron remnants. Because they carry apoB and lack apoE, we refer to them as apoB-containing, apoE-free (B^+^/E^-^) lipoproteins.

### Cholesterol Efflux Assay

The effect of R5-6 on cholesterol efflux was studied using purified R5-6 protein and R5-6-conditioned medium. Specifically, RAW 264.7 cells grown in 24-well plates at confluence were incubated with 30 μg/ml of mouse apoB-carrying lipoproteins and 0.25 μCi/ml of ^3^H-cholesterol for 18 h, as described previously [15]. In the experiments using purified R5-6 protein, radiolabeled cells were treated with 5 μg/ml of c-myc peptide, 0.2 μg/ml of R5-6 or vehicle control in the presence or absence of 20 μg/ml of apoAI for 3 h. In the experiments using conditioned medium, radiolabeled cells were incubated with control or R5-6-conditioned medium in the presence or absence of 20 μg/ml of apoAI for 3 h. In the experiments using PI3K and Sp1 inhibitors, 50 μM of LY294002 or 100 nM mithramycin A or ethanol vehicle to 0.1% was added into the medium. The culture medium was collected, and the cell pellet was lysed with 10 volumes of cell lysis buffer (50 mM NaCl, 0.5% Triton X-100, and 20 mM Tris-HCl, pH 7.4). The lysate and medium were each mixed with scintillation fluid to assay radioactivity using a Tri-Carb 2300TR Liquid Scintillation Analyzer (Perkin Elmer). Cholesterol efflux in the cells treated with or without apoAI was expressed as the percentage of radioactivity in the medium compared to the total radioactivity (cells plus medium). The apoAI-mediated cholesterol efflux was calculated by subtracting the efflux in the absence of apoAI from the efflux in the presence of apoAI.

### Cellular Cholesterol Content Measurement

Confluent RAW264.7 cells were incubated with control, R5-6- or R5-6C-conditioned medium ± 30 μg/ml of B^+^/E^-^ lipoproteins for 72 h. Total lipids were extracted with chloroform: methanol (2:1) and the solvent was evaporated under vacuum [14,26]. The pellet was resuspended in cholesterol assay buffer (100 mM KH_2_PO4, pH 8.0 with KOH, 50 mM KCl, and 10 mM cholic acid). Free and total cholesterol were measured enzymatically using cholesterol assay reagents from Wako Diagnostics. The amount of esterified cholesterol was calculated as the difference of the total cholesterol and free cholesterol.

### Small Interfering RNA (siRNA) Knockdown of VLDLR and ApoER2

RAW 264.7 cells were transfected with scrambled siRNA or specific siRNAs against VLDLR or apoER2 using Fugene HD reagent and OptiMEM medium according to the manufacturer's instructions. After 6 h, cells were replenished with fresh medium containing 10% FBS and cultured for an additional 24 h. These transfection procedures were repeated one more time [27]. The knockdown efficiency induced by siRNA was confirmed by detection of VLDLR and apoER2 mRNAs and proteins using real-time RT-PCR and western blot analysis [15], respectively. We observed that the optimal knockdown of apoER2 and VLDLR occurred within 48 h (data not shown). The transfected cells were incubated with the control- or R5-6-conditioned medium ± 30 μg/ml B^+^/E^-^ lipoproteins for 48 h, and cellular cholesterol content was measured as described above.

### Quantitative Real-Time RT-PCR Assay

Total RNA was extracted from Raw 264.7 cells using Trizol reagent, DNAse I-treated, and subjected to reverse transcription using a high capacity cDNA reverse transcription kit (Applied Biosystems). The resulting cDNAs were subjected to quantitative real-time RT-PCR with an iCycler system (Bio-Bad) using primers synthesized by Qiagen for amplification of VLDLR, apoER2, ABCA1 and glyceraldehyde 3-phosphate dehydrogenase (GAPDH) [28]. The following specific primers were used for amplification: apoER2 forward: 5’-TGTCCTGCCGAGAAGTTAAG-3’, reverse: 5’-ACCCTCACAGTCCTTCTCTC-3’; VLDLR forward: 5’-TGAAAGCCCTGAACAGTGCC-3’, reverse: 5’- AGTCATCCTGGCCATTGCAC-3’; ABCA1 forward: 5’-GCTACCCACCCTACGAACAA-3, reverse: 5’-GGAGTTGGATAACGGAAGCA-3’; and GAPDH forward: 5’-GAGCCAAAAGGGTCATCATC-3’, reverse: 5’-TAAGCAGTTGGTGGTGCAGG-3’.

### Western Blot Analysis

In the experiments for studying protein secretion, medium harvested from empty vector, R5-6, or R5-6C transfected cells was mixed with methanol to a final concentration of 60%, and incubated at -20°C for 1 h, followed by centrifugation at 16000 x g for 2 min at 4°C. The pellet was resuspended in urea loading buffer (9 M urea, 125 mM Tris-HCl, pH 6.8, 715 mM β-mercaptoethanol, 2% SDS). In the experiments for studying the effect of reelin subregions on protein expression, RAW 264.7 cells were equilibrated in DMEM for 3 h, then incubated with DMEM ± 0.2 μg/ml of purified R5-6, or with control-, R5-6- or R5-6C-conditioned medium for 3 h. In the experiments involving PI3K and Sp1 inhibitors, 50 μM LY294002 or 100 nM mithramycin A were added into the medium. The cells were lysed in M-PER mammalian protein extraction reagent. Cell lysates and the proteins precipitated from the culture medium were resolved on 10% SDS-PAGE gels. Proteins were transferred to a PVDF membrane. After blocking with 3% fat-free milk, membranes were incubated with antibodies as indicated in the figure legends. Immunoreactive bands were visualized using ECL-plus chemiluminescence reagent (GE Healthcare–Amersham) and analyzed with a GS-700 Imaging Densitometer (Bio-Rad) [28].

### Statistical Analysis

Data are reported as the mean ± the standard error of the mean of at least four independent experiments. Differences among control and treatment groups were analyzed by Student’s unpaired *t*-test (for two groups) and one-way or multiple factor analysis of variance (for more than two groups) followed by Tukey’s post-hoc test. Statistical significance was considered when *P* was less than 0.05. VassarStats (vassarstats.net) software was used for statistical analysis.

## Results

### Both R5-6 and R5-6C are Secretable and Able to Bind Receptors

**Fig 1:**
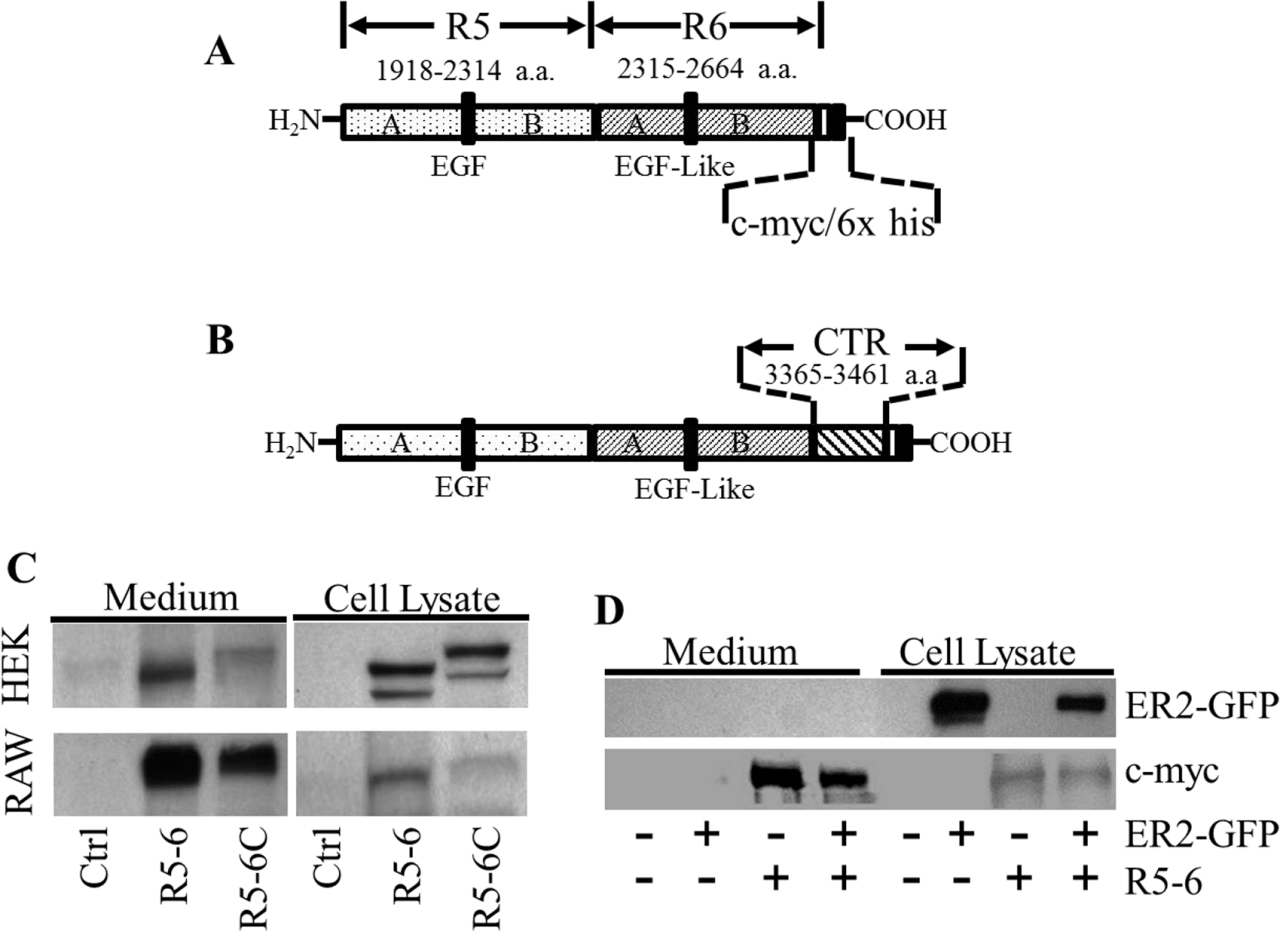
The secretability of reelin subregions. (A) Reelin subregion R5-6 containing the 5^th^ and 6^th^ repeats of reelin. The numbers represent the amino acid position in the full-length reelin. Epidermal growth factor (EGF) and EGF-like motifs are indicated with vertical bars. The fragment terminates with a c-myc epitope and a 6x histidine tag. (B) Reelin subregion R5-6C containing the 5^th^ and 6^th^ repeats and the C-terminal region (CTR) of reelin. The diagrams are not drawn to scale. (C) HEK293 and RAW 264.7 cells were transfected with the indicated reelin constructs or empty pcDNA3.1B vector as a control (Ctrl), and the proteins were detected by western blotting using c-myc epitope antibody. (D) RAW 264.7 cells were transfected with empty pcDNA3.1B vector, R5-6, apoER2-GFP (ER2-GFP) expression plasmid, or co-transfected with R5-6 and ER2-GFP expression plasmids. R5-6, R5-6C and ER2-GFP fusion protein in the medium and cell lysate were detected by using antibodies against c-myc epitope and apoER2, respectively.

The primary structures of R5-6 and R5-6C are shown in Fig 1A and 1B, respectively. Reelin repeat 5 (R5) contains 397 amino acids, while repeat 6 (R6) contains 350 amino acids. An epidermal growth factor (EGF) or EGF-like motif divides each of R5 and R6 into A and B subrepeats. R5-6C includes the last 97 amino acids at the c-terminus of reelin, ending with the well-conserved 32 amino acids [29]. R5-6 and R5-6C constructs were predicted to produce two proteins of ~88 and ~100 kd, respectively. The image in Fig 1C shows a single protein band for R5-6 or R5-6C in RAW 264.7 cells, but two bands for each of them in HEK293 cells, with a stronger intensity for the top band. The immunoblotting bands of RAW 264.7 cells and the top bands of HEK293 cells were consistent with the sizes of R5-6 and R5-6C proteins predicted from their amino acid sequences. The bottom immunoblotting bands of HEK293 cells were smaller than the predicted sizes of R5-6 or R5-6C. These suggest that post-translational modification of R5-6 and R5-6C in HEK 293 cells differs from that in RAW 264.7 cells. The physiological significance of these modifications is unknown.

Fig 1C also shows that R5-6 and R5-6C proteins were detectable in both cell lysates and culture media. These bands represent authentic R5-6 and R5-6C, as demonstrated by their expected positions detected only in transfected cells. This suggests that R5-6 and R5-6C can be secreted by cells. However, the secretion of reelin subregion proteins from RAW 264.7 cells appears to be greater than from HEK 293 cells. Specifically, about 90 and 60% of R5-6 and R5-6C were, respectively, found in the medium cultured with RAW264.7 cells, while only about 30 and 20% of these partial reelin proteins were found in the medium cultured with HEK293 cells (Fig 1C). These results imply that R5-6 can be secreted from cells in the absence of the CTR, and that the secretion rate of R5-6 and R5-6C is cell-specific.

To confirm the secretability of R5-6, we transfected RAW 264. 7 cells with R5-6 and apoER2-GFP expression plasmids together or separately, and then detected the proteins in cells and medium. The images in Fig 1D show that R5-6 protein was present in both the cells transfected with R5-6 constructs and the medium incubated with these cells. In contrast, the apoER2-GFP fusion protein was present only in cells transfected with apoER2-GFP expression plasmids, but barely detectable in the medium incubated with these cells. These results suggest that RAW 264. 7 cells are able to secrete R5-6 but cannot secrete the apoER2-GFP fusion protein into culture medium, and that the R5-6 protein in the medium does come from an intact cellular process rather than dying cells.

**Fig 2:**
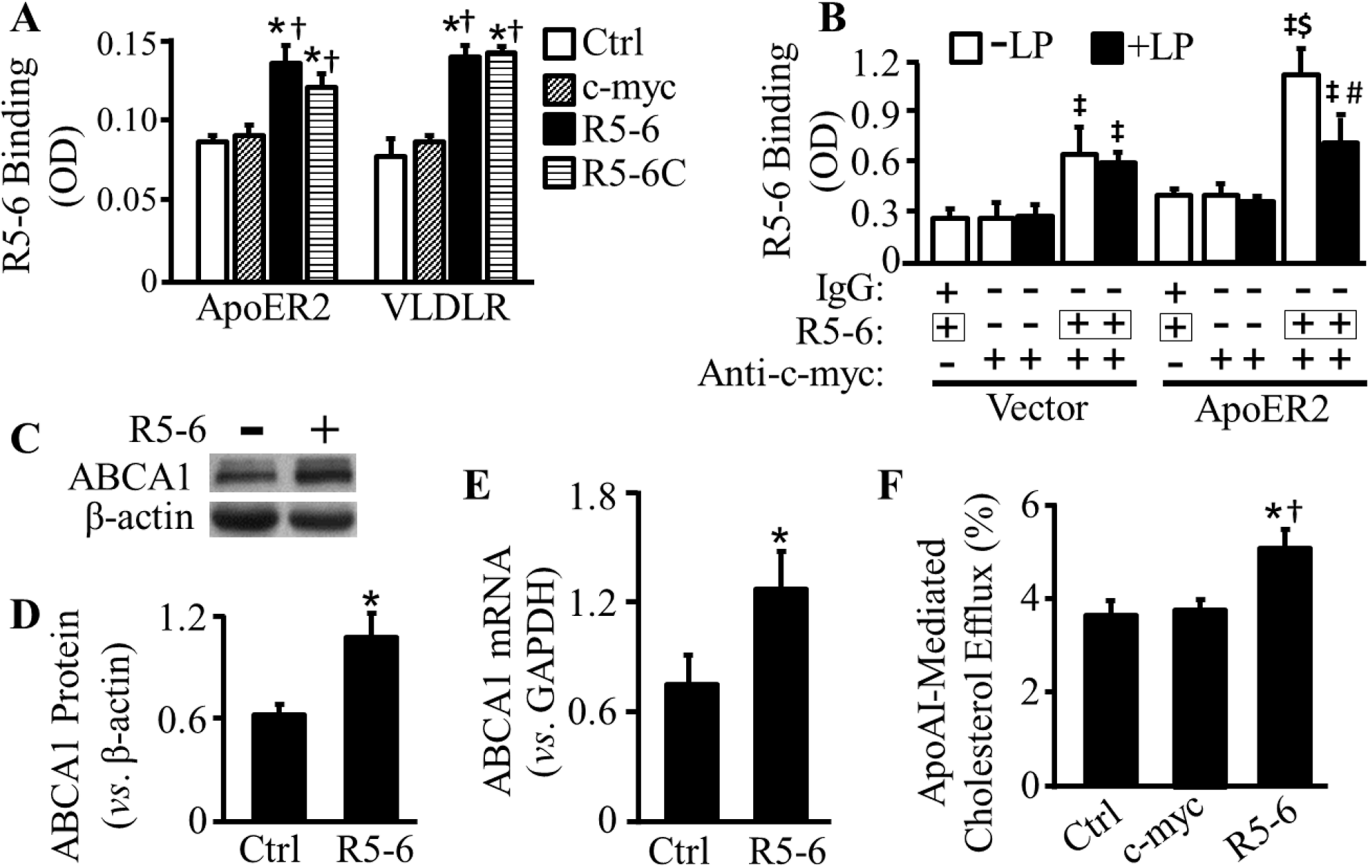
The ability of purified reelin subregions to bind apoER2 and VLDLR and upregulate ABCA1 expression. (A) ELISA plates were coated with c-myc peptide, purified R5-6 or R5-6C, or null eluent from empty-vector transformed bacteria as a control (Ctrl), and then incubated with RAW 264.7 cell lysates. The apoER2 and VLDLR in RAW 264.7 cell lysates bound to ELISA plates were detected using antibodies against apoER2 and VLDLR. (B) RAW 264.7 cells were transfected with empty pcDNA3.1B vector or apoER2-GFP expression plasmid, and then incubated with 30 μg/ml of apoB-carrying and apoE-free lipoproteins (+LP) or without lipoproteins (-LP) in the presence or absence of 0.2 μg/ml purified R5-6 protein. The amount of R5-6 bound to the cell surface was determined by ELISA using an antibody against c-myc epitope. The background binding was determined using a normal mouse IgG. (C-D) RAW 264.7 cells were treated with 0.2 μg/ml of purified R5-6 protein or culture medium alone (Ctrl). The protein level of ABCA1 was determined by immunoblotting and quantified relative to β-actin. (E) RAW 246.7 cells were treated as described in plane B. The mRNA level of ABCA1 was determined by quantitative real-time RT-PCR and normalized relative to GAPDH mRNA. (F) RAW 264.7 cells were labeled with 0.25 μCi/ml of ^3^H-cholesterol, followed by incubation with 5 μg/ml c-myc peptide, 0.2 μg/ml purified R5-6 protein or vehicle control (Ctrl). Cholesterol efflux was determined in cells incubated with or without apoAI treatment, and was expressed as the percentage of radioactivity in the medium compared to the total radioactivity in the cells and medium. ApoAI-mediated cholesterol efflux was calculated as the difference of efflux from cells in the presence and absence of apoAI treatment. Data represent the mean ± SEM of four or more independent experiments. * *p*< 0.05 *vs*. Ctrl; ^†^, *p*<0.05 *vs*. c-myc-immobilized ELISA plates or treated cells; ^‡^, *p*< 0.05 *vs*. cells transfected with the same plasmids and untreated with R5-6; ^$^, *p*< 0.05 *vs*. cells transfected with empty vector and treated with R5-6; and ^#^, *p*< 0.05 *vs*. cells transfected with apoER2-GFP-expression plasmid and untreated with lipoproteins.

Previous reports determined the receptor binding activity of R5-6 by solid-phase binding assay, in which agarose beads bound with VLDLR or apoER2 ectodomain, and cell lysates derived from R5-6-overexpressing cells were used [19,20]. Data from those studies are inconsistent. Specifically, one of the reports showed that R5-6 failed to bind the ectodomains of apoER2 and VLDLR [19], while another study showed that R5-6 can bind the ectodomains of apoER2 [20]. In the present study, we determined the ability of R5-6 or R5-6C to bind apoER2 and VLDLR using a protein immobilization-based sandwich ELISA. As Fig 2A shows, the binding of apoER2 and VLDLR to the R5-6- or R5-6C-immobilized plates was increased by ~50–80% as compared to the control plates. The purified R5-6 protein is fused with a c-myc epitope tag. The data in Fig 2A show that c-myc peptide did not bind apoER2 nor VLDLR as judged from the eluent control baseline.

We also determined the ability of R5-6 to bind the surface of RAW 264.7 macrophages with or without overexpression of apoER2. As can be seen in Fig 2B, R5-6 protein is able to bind to the cell surface of macrophages. The amount of R5-6 bound to the apoER2-overexpressing cells was elevated by ~74%, as compared to the cells without apoER2 overexpression. Fig 2B also shows that the addition of B^+^/E^-^ lipoproteins to the culture medium diminished the amount of R5-6 bound to the apoER2-overexpressing cells by 37%, though the lipoproteins did not significantly reduce the amount of bound R5-6 in the cells transfected with the control vector. These results suggest that B^+^/E^-^ lipoproteins are able to compete with R5-6 to bind apoER2.

### R5-6 Upregulates ABCA1 expression and Enhances ApoAI-Mediated Cholesterol Efflux

**Fig 3:**
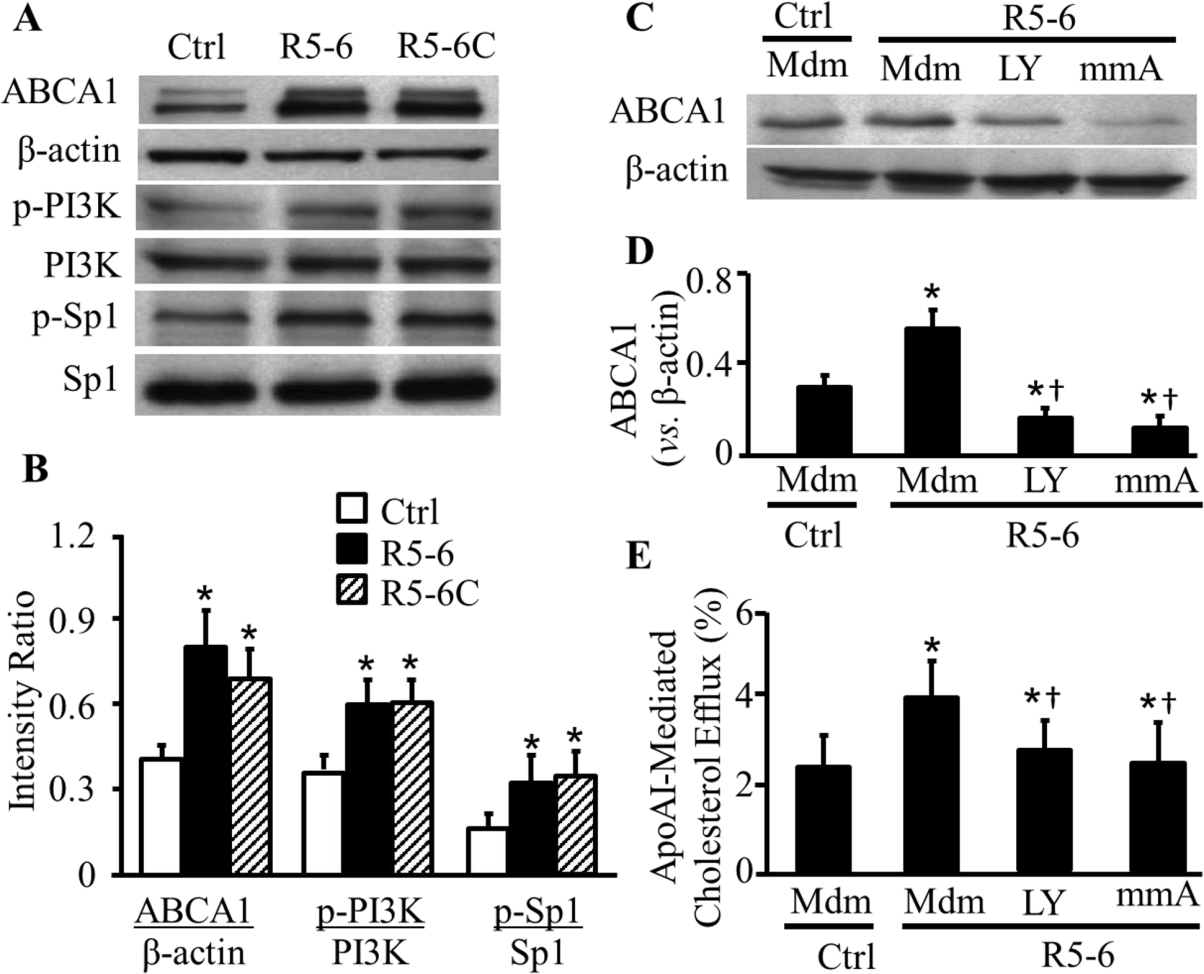
Effect of reelin subregions on ABCA1, p-PI3K, and p-Sp1 levels. (***A-B***) RAW 264.7 cells were transfected with empty pcDNA3.1B vector, or plasmids carrying reelin subregions R5-6 or R5-6C to generate control (Ctrl), R5-6- or R5-6C-conditioned medium. Untransfected RAW 264.7 cells were incubated with Ctrl, R5-6- or R5-6C-conditioned medium for 3 h. The indicated proteins were measured by immunoblotting. The representative images (A) were generated with a single experiment using cell lysates loaded in multiple gels. The level of ABCA1 was quantified relative to β-actin, and the level of phosphorylated Sp1 (p-Sp1) and PI3K (p-PI3K) was expressed relative to total Sp1 and PI3K, respectively. (C-D) Untransfected RAW 264.7 cells were incubated with Ctrl or R5-6-conditioned medium (Mdm) ± 100 nM mithramycin A (mmA) or 50 μM LY294002 (LY) for 3 h. The level of ABCA1 was determined by immunoblotting and quantified relative to β-actin. (E) Untransfected RAW 264.7 cells were labeled with 0.25 μCi/ml of ^3^H-cholesterol, followed by incubation with Control or R5-6-conditioned medium ± 15 μg/ml of apoAI, 100 nM mmA or 50 μM LY. Cholesterol efflux was determined in cells incubated with or without apoAI treatment, and was expressed as the percentage of radioactivity in the medium compared to the total radioactivity in the cells and medium. ApoAI-mediated cholesterol efflux was calculated as the difference of efflux from cells in the presence and absence of apoAI treatment. Data represent the mean ± SEM of four or more independent experiments. *, *p*< 0.05 *vs*. control conditioned medium; and ^†^, *p*< 0.05 *vs*. cells treated with R5-6-conditioned medium alone.

We previously reported that the full-length reelin central fragment (R3-6) is able to upregulate ABCA1 expression and enhance apoAI-mediated cholesterol efflux [15]. In this study, we determined the effect of R5-6 on macrophage ABCA1 expression and cholesterol efflux. Data in Fig 2C–2E show that incubation of RAW 264.7 cells with 0.2 μg/ml of purified R5-6 protein for 3 h increased ABCA1 protein level by 72% and ABCA1 mRNA level by 70%. The data in Fig 3A and 3B suggest that cell-secreted reelin subregions have a similar up-regulatory effect on ABCA1 expression as the purified R5-6 protein. Specifically, incubation of RAW 264.7 cells with R5-6- or R5-6C-conditioned medium for 3 h increased ABCA1 protein level by 96 and 69%, respectively, compared to cells incubated with the control conditioned medium (Fig 3A and 3B).

ABCA1 is a cell membrane protein that mediates cholesterol efflux [30]. Lipid-free apoAI reportedly serves as an acceptor for ABCA1-mediated cholesterol efflux [30]. We therefore studied the effect of R5-6 on apoAI-mediated cholesterol efflux. As shown in Figs 2F and 3E, treatment of RAW 264.7 cells with 0.2 μg/ml of purified R5-6 protein or R5-6-conditioned medium increased apoAI-mediated cholesterol efflux by ~1.39 and 1.68 fold, respectively. Namely, apoAI-mediated cholesterol efflux was increased from ~3.6% to ~5% in cells treated with purified R5-6 protein (Fig 2F), and increased from ~2.3% to ~4% in cells treated with R5-6-conditioned medium (Fig 3E), as compared to the vehicle control or the control conditioned medium, respectively. Treatment of with c-myc peptide did not increases cholesterol efflux in RAW 264.7 cells (Fig 2F).

### Activation of PI3K and Sp1 Is a Mechanism for R5-6-Induced ABCA1 Expression and Cholesterol Efflux

Activations of PI3K and Sp1 are key steps in the signaling cascade through which VLDLR and apoER2 upregulate ABCA1 expression and accelerate cholesterol efflux [15]. Herein we studied the involvement of PI3K and Sp1 in R5-6-induced ABCA1 expression and cholesterol efflux. Sp1 is one of the transcription factors that controls ABCA1 expression [31]. An increase in the phosphorylation of Sp1 enhances its capacity to bind to the ABCA1 promoter and upregulate ABCA1 expression [31]. Activation of PI3K, on the other hand, is a step in the signaling cascade that transduces signaling from VLDLR/apoER2 to Sp1. As Fig 3A and 3B shows, the R5-6- and R5-6C-conditioned media enhanced phosphorylation of PI3K and Sp1 by ~65 and 97%, respectively; but did not alter the total PI3K and Sp1 protein levels.

Having established the regulatory role of R5-6 in ABCA1 expression, PI3K and Sp1 phosphorylation, we next studied the impact of these signaling proteins on ABCA1 expression by using previously reported inhibitors [31]. LY294002 is a PI3K inhibitor [32], while mithramycin A is a chemotherapeutic drug that binds to GC-rich DNA sequences, thereby blocking the binding of transcription factors such as Sp1 to GC-specific regions of DNA [33]. In this study, RAW 264. 7 cells were incubated with R5-6-conditioned medium in the presence of these inhibitors. The data in Fig 3C and 3D indicate that either LY294002 or mithramycin A abolished the upregulatory activity of R5-6-conditioned medium on ABCA1 protein expression, *i*.*e*., R5-6-conditioned medium did not significantly elevate ABCA1 protein level in cells treated with these inhibitors.

To ascertain the regulatory role of PI3K and Sp1 in R5-6-induced cholesterol efflux, we studied the impact of inhibiting PI3K and Sp1 on cholesterol efflux. As can be seen in Fig 3E, treatment of macrophages with LY294002 or mithramycin A diminished cholesterol efflux induced by R5-6., *i*.*e*., incubation of RAW264.7 cells with R5-6-conditioned medium did not significantly elevate cholesterol efflux in cells treated with either one of these inhibitors.

### R5-6 Inhibits Cellular Cholesterol Accumulation by Activation of VLDLR and ApoER2

**Fig 4:**
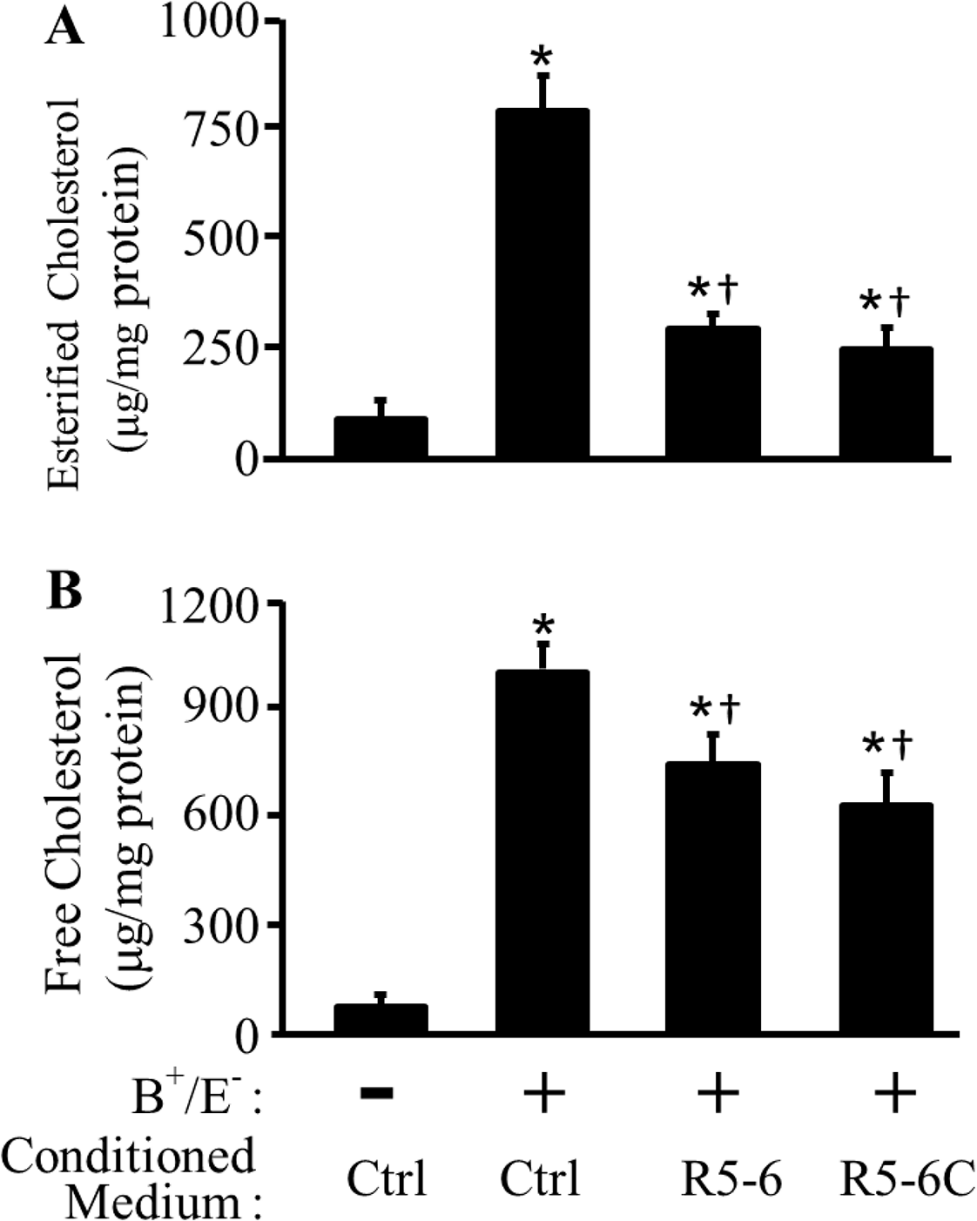
Effect of reelin subregions on macrophage cholesterol accumulation. RAW 264.7 cells were transfected with empty pcDNA3.1B vector or plasmids carrying reelin subregions R5-6 or R5-6C to generate control (Ctrl), R5-6- or R5-6C-conditioned medium. Untransfected RAW 264.7 cells were incubated with Ctrl, R5-6- or R5-6C-conditioned medium ± 30 μg/ml of apoB-carrying and apoE-free (B^+^/E^-^) lipoproteins for 72 h. Lipids were extracted from the cells for determination of total and free cholesterol. The amount of esterified cholesterol was calculated as the difference of the total cholesterol and free cholesterol. Data represent the mean ± SEM of four independent experiments. *, *p* < 0.05 *vs*. cells without B^+^/E^-^ lipoprotein treatment; and ^†^, *p* < 0.05 *vs*. cells treated with B^+^/E^-^ lipoproteins in the control conditioned medium.

Accumulation of cellular cholesterol, especially esterified cholesterol, is a hallmark of foam cells. Given that efflux of excessive cholesterol is a mechanism that prevents cellular cholesterol accumulation, we determined the effect of R5-6 and R5-6C on cellular cholesterol content. As Fig 4A and 4B show, treatment of RAW 264.7 cells with B^+^/E^-^ lipoproteins for 72 h increased esterified cholesterol (EC) and free cholesterol (FC) by ~8 and 12 fold, respectively. These agree with previous studies showing that B^+^/E^-^ are able to transform macrophages into foam cells [34]. The data in Fig 4 also demonstrate that treatment of RAW 264.7 cells with R5-6- or R5-6C-conditioned medium diminished B^+^/E^-^ lipoprotein-induced cholesterol accumulation. Specifically, the EC levels in the cells incubated with R5-6- or R5-6C-conditioned medium were ~63 and 69% lower, respectively, than in cells incubated with the control conditioned medium in the presence of B^+^/E^-^ lipoproteins (Fig 4A). The R5-6- and R5-6C-conditioned media also reduced the FC by ~26 and 37% in RAW 264.7 cells, respectively (Fig 4B).

**Fig 5:**
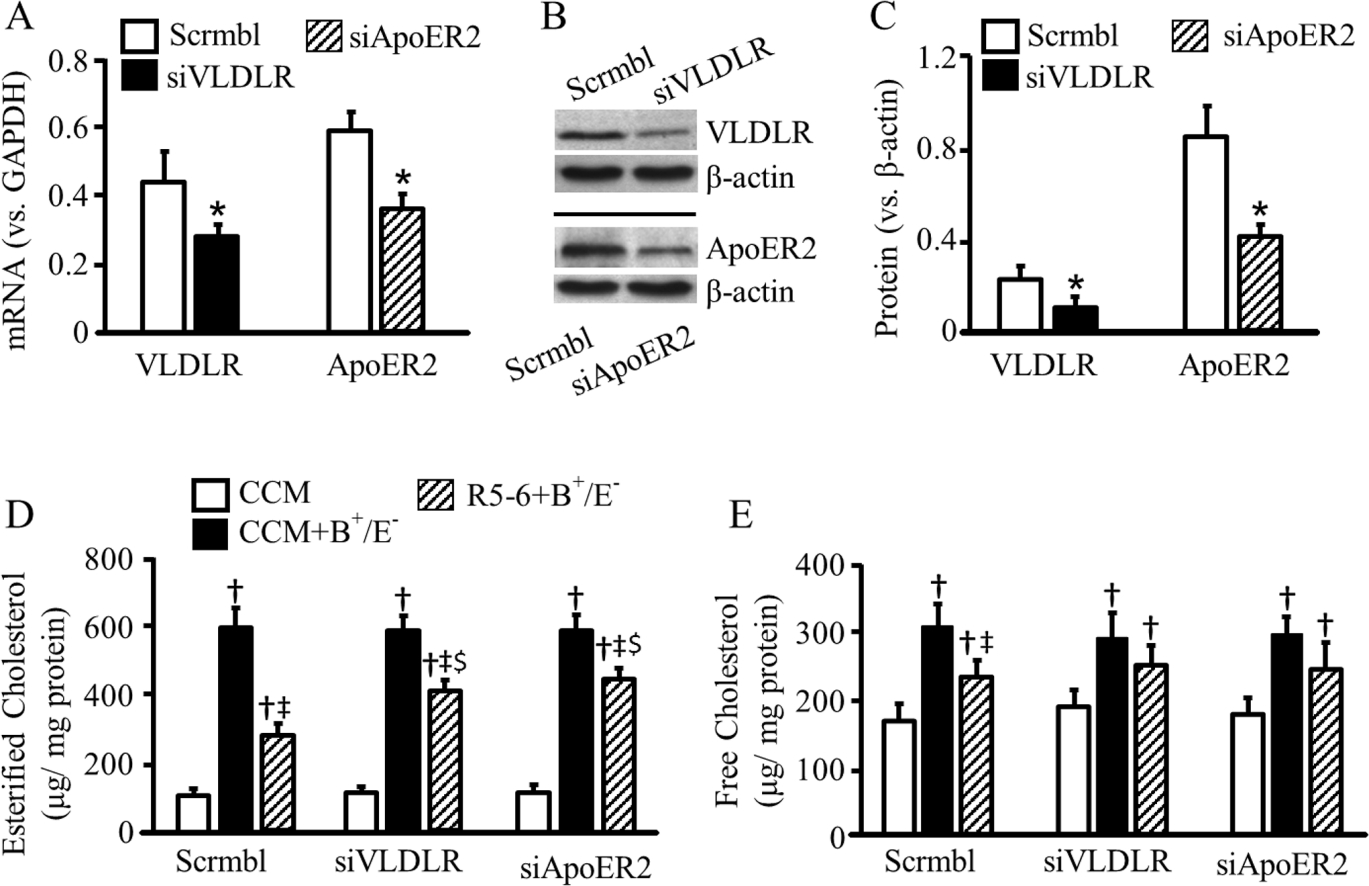
Knockdown of VLDLR and apoER2 attenuates the protective effect of reelin subregion R5-6 against macrophage cholesterol accumulation. (***A-C***) RAW 264.7 cells were transfected with siRNA specific for VLDLR (siVLDLR) or apoER2 (siApoER2), or scrambled siRNA (scrmbl). The mRNA levels of VLDLR and apoER2 were determined by quantitative real-time RT-PCR and normalized to GAPDH mRNA (A). The protein levels of VLDLR and apoER2 were determined by western blot analysis and normalized to β-actin (B-C). (D-E) RAW 264.7 cells were transfected with empty pcDNA3.1B vector or plasmids carrying reelin subregion R5-6 to generate control conditioned medium (CCM) or R5-6-conditioned medium. The scrambled siRNA-, siVLDLR- or siApoER2-transfected cells were incubated with CCM or R5-6-conditioned medium (R5-6) ± 30 μg/ml of apoB-containing, apoE-free (B^+^/E^-^) lipoproteins for 48 h. Lipids were extracted from the cells for determination of total and free cholesterol. The amount of esterified cholesterol was calculated as the difference of the total cholesterol and free cholesterol. Data represent the mean ± SEM of 5–7 independent experiments. *, *p <* 0.05 *vs*. cells transfected with scrambled siRNA; ^†^, *p* <0.05 *vs*. cells transfected with the same siRNA and treated with control conditioned medium; ^‡^, *p* <0.05 *vs*. cells transfected with the same siRNA and treated with control conditioned medium plus B^+^/E^-^ lipoproteins; and ^$^, *p*<0.05 vs. cells transfected with scrambled siRNA and treated with R5-6-conditioned medium plus B^+^/E^-^ lipoproteins.

To address the involvement of apoER2 and VLDLR in R5-6-induced protection against cellular cholesterol accumulation, apoER2 and VLDLR expression were down-regulated by siRNAs. The knockdown efficiency induced by siRNA was confirmed by detection of VLDLR and ApoER2 mRNAs and proteins (Fig 5A–5C). The expression level of VLDLR was lower than apoER2 in RAW264.7 cells. Transfection of these cells with siRNA against apoER2 or VLDLR reduced the mRNA and protein levels of apoER2 by ~39 and 52%, and reduced the mRNA and protein levels of VLDLR by ~36 and 52%, respectively (Fig 5A–5C).

The data in Fig 5D and 5E show that the cholesterol content was comparable in RAW264.7 cells transfected with VLDLR siRNA, apoER2 siRNA or scrambled siRNA in the absence of B^+^/E^-^ lipoprotein treatment. Incubation of these cells with 30 μg/ml of B^+^/E^-^ lipoproteins in the control conditioned medium for 48 h increased EC and FC by 5.0–5.5 and 1.4–1.6 fold, respectively. There was no significant difference among cells transfected with different siRNAs. These data suggest that knockdown of VLDLR or apoER2 in macrophages did not affect the basal cholesterol content and the sensitivity to B^+^/E^-^ lipoprotein-induced cholesterol accumulation. In contrast, B^+^/E^-^ lipoprotein-induced cholesterol accumulation in the presence of R5-6 varied among cells transfected with different siRNAs. Specifically, incubation of the scrambled siRNA-transfected cells with 30 μg/ml of B^+^/E^-^ lipoproteins for 48 h in R5-6-conditioned medium increased EC and FC by 2.5 and 1.2 fold, respectively; while B^+^/E^-^ lipoproteins under the same culture condition increased EC and FC by 3.5 and 1.2 fold in apoER2 siRNA-transfected cells, and 4 and 1.3 fold in VLDLR siRNA-transfected cells, respectively. The B^+^/E^-^ lipoproteins-induced EC accumulation was significantly higher in cells transfected with apoER2 and VLDLR siRNAs than in those transfected with scrambled siRNA.

## Discussion

Both VLDLR and apoER2 are able to mediate the endocytosis of native and modified lipoproteins [2,8,35–37]. Unrestricted uptake of these lipoproteins via VLDLR and apoER2 has been suggested to induce macrophage cholesterol accumulation and accelerate foam cell formation ^28–30^. In contrast to this note, data from the current report suggest that activation of VLDLR and apoER2 by reelin subregion R5-6 inhibits cholesterol accumulation and foam cell formation. Specifically, we observed that treatment of mouse macrophages with R5-6 significantly reduced B^+^/E^-^ lipoprotein-induced cholesterol accumulation. Knockdown of VLDLR or apoER2 attenuated the protective role of R5-6 against B^+^/E^-^ lipoprotein-induced cholesterol accumulation. These findings imply that the pro-atherogenic effect of VLDLR and apoER2 arising from the lipoprotein uptake function could be counterbalanced by the anti-atherogenic effects induced by R5-6.

Data from this report also show that R5-6 was able to increase ABCA1 expression and cholesterol efflux in macrophages. ABCA1 is a key protein in the removal of excess cellular cholesterol [38–41]. Increasing evidence clearly indicates an anti-atherogenic role for ABCA1-mediated cholesterol efflux in macrophages [38–42]. Specifically, mutation of ABCA1 in humans has been shown to induce cholesterol accumulation in cells of the reticuloendothelial system, and increase susceptibility to atherosclerosis [42]. Knockout of ABCA1 gene in leukocytes worsens the progress of atherosclerosis in LDL receptor knockout (*LDLR* ^*-/-*^) mice [38]. On the other hand, overexpression of ABCA1 retards signs of atherosclerosis in mice [40,41]. Likewise, macrophages that overexpress ABCA1 are efficient in preventing cholesterol accumulation [40]. Considering the protective role of ABCA1 against cholesterol accumulation, it would be rational to postulate that increase in ABCA1 expression and ABCA1-mediated cholesterol efflux is a mechanism by which R5-6 reduces B^+^/E^-^ lipoprotein-induced cholesterol accumulation in macrophages.

Previous works from our laboratory suggest that the up-regulatory activity of VLDLR and apoER2 on ABCA1 expression is mediated through a signaling cascade involving Dab1, PI3K, and protein kinase C-ζ (PKC-ζ), which activates transcription factors Sp1 and liver X receptor (LXR)/ [13–15,31]. This report selectively studied the causative role of PI3K and Sp1 on R5-6-induced ABCA1 expression. We previously observed that the increase in ABCA1 mRNA expression induced by B^+^/E^-^ lipoproteins, lipid-poor apoE or the full length reelin central fragment R3-6 was associated with an enhanced phosphorylation of PI3K and Sp1, and increased binding Sp1 to the ABCA1 promoter region [13–15,31]. Knockdown of VLDLR or apoER2 attenuated PI3K phosphorylation induced by B^+^/E^-^ lipoproteins, apoE or R3-6. Inhibition of PI3K attenuated the phosphorylation of Sp1 induced by these ligands [13–15,31]. Mutation of the Sp1 and LXR binding motif in the ABCA1 promoter region or inhibition of Sp1-DNA binding by mithramycin A diminished the inducibility of ABCA1 promoter by B^+^/E^-^ lipoproteins and apoE [13,31]. Knockdown of VLDLR and apoER2, or inhibition of PI3K and Sp1 diminished ABCA1 mRNA expression induced by B^+^/E^-^ lipoproteins, apoE, or R3-6 [13–15,31]. In the present report, we demonstrated that treatment of macrophages with R5-6-conditioned medium enhanced PI3K and Sp1 phosphorylation, and inhibition of PI3K and Sp1 reduced R5-6-induced ABCA1 expression. These findings suggest that activation of PI3K and Sp1 could be a mechanism by which R5-6 up-regulates ABCA1 expression.

The present report did not study the signaling transduction between VLDLR/apoER2 and PI3K, as well the transduction between PI3K and Sp1. However, it has been well established that activation of VLDLR and aopER2 induces tyrosine phosphorylation of the cytoplasmic adaptor protein Dab1 [11,43], which in turn activates PI3K. Activated PI3K phosphorylates phosphoinositides to form phosphotidylinositol 3-phosphate and other phosphorylated phosphoinositides. These lipids bind phosphoinositide-dependent protein kinase 1 (PDK1) and other signaling proteins, such as Akt and PKC ζ. This co-localization allows PDK1 to phosphorylate Akt [44] and PKC ζ [45]. We previously observed that besides induction of PI3K and Sp1 phosphorylation, apoE and B^+^/E^-^ lipoproteins also enhanced Dab1 and PKC ζ phosphorylation, and induced physical interaction of PKC-ζ and Sp1. Knockdown of Dab1 diminished apoE-induced PI3K phosphorylation, inhibition of PI3K reduced apoE-induced PKC-ζ phosphorylation [31], while inhibition of PKC-ζ attenuated apoE-induced Sp1 phosphorylation. Inhibition of Dab1, PI3K, PKC-ζ and Sp1 reduced ABCA1 expression or inhibited ABCA1 promoter activity. Taken together, it is highly likely that activation of VLDLR and apoER2 sequentially triggers Dab1, PI3K, PDK1 and PKC-ζ, which in turn phosphorylates Sp1, increasing its binding to the ABCA1 promoter and up-regulating ABCA1 transcription. Further studies are required to confirm the involvement of Dab1, PDK1, and PKC-ζ in the R5-6-induced ABCA1 expression.

Reelin is a secreted glycoprotein. The mechanism for secretion of the full length of reelin protein has not been fully defined. The role of the CTR for reelin secretion is under debate, *i*.*e*., the CTR was reported to be either essential [21] or dispensable [22] for release of the full length reelin protein from cells. Here we demonstrate that both R5-6C and R5-6 can be secreted from cells, and suggest that release of the reelin subregion R5-6 from cells does not require the CTR. It is of interest to note that the secretion of R5-6 is greater in RAW264.7 cells than in HEK 293. The mechanism for the variation of R5-6 secretion in different cells remains unknown. The secretability of R5-6 would be important for future *in vivo* gene transfer studies. It is anticipated that R5-6 overexpressed in cells could be secreted, interact with VLDLR and/or apoER2 in endothelial cells, smooth muscle cells and macrophages, and play anti-atherogenic roles.

In summary, this report demonstrated that the reelin subregion R5-6 consisting of 747 amino acids in the 5^th^ and 6^th^ repeats was sufficient for binding apoER2 and VLDLR, and for inhibiting lipoprotein-induced cholesterol accumulation in macrophages. Knockdown of VLDLR or apoER2 attenuated the protective role of R5-6 against intracellular cholesterol accumulation. In addition, R5-6 activated Sp1 and PI3K, up-regulated ABCA1 expression, and accelerated cholesterol efflux. These data suggest that R5-6 can function as an anti-atherogenic ligand of VLDLR and apoER2. It is highly likely that binding of R5-6 to VLDLR and apoER2 activates a pathway involving PI3K and Sp1, which up-regulates ABCA1 expression and accelerates cholesterol efflux, and therefore inhibits cholesterol accumulation. In addition, data from this report suggest that R5-6 and B^+^/E^-^ lipoproteins compete to bind apoER2. It has been reported that apoB100 is a ligand of apoER2 [8,9]. It is thus possible that binding of R5-6 to apoER2 forestalls the binding of the apoB100 component of B^+^/E^-^ lipoproteins to this receptor. Inhibition of apoB100 binding to apoER2 might reduce the endocytosis of apoB100-carrying lipoproteins, and therefore reduce cellular cholesterol accumulation. Further studies are required to determine the impact of R5-6 on macrophage uptake of lipoproteins. As the full length reelin (~400-kd) or its central fragment R3-6 (340 kd) is too large to be used in gene transfer studies, the reelin subregion R5-6 (~88kd) could be a valuable tool in future studies for investigating the effect of VLDLR/apoER2 on atherosclerosis in animal models.

## Supporting Information

S1 ARRIVE Checklist (DOCX)
