# Supplementary material for: A Subregion of Reelin Suppresses Lipoprotein-Induced Cholesterol Accumulation in Macrophages
Source: PLoS One. 2015 Aug 28;10(8):e0136895. doi: 10.1371/journal.pone.0136895 (PMC4552883; doi:10.1371/journal.pone.0136895)

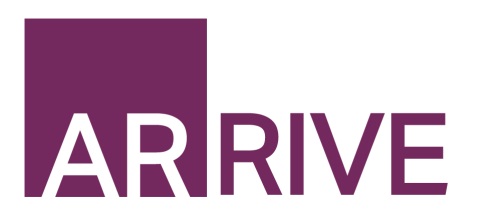


The ARRIVE Checklist

A Subregion of Reelin Suppresses Lipoprotein-Induced Cholesterol Accumulation in Macrophages

Emmanuel U. Okoro^1,¶^, Hongfeng Zhang^1,2,¶^, Zhongmao Guo^1^, Fang Yang^1,3^, Carlie Smith Jr.^1^ and Hong Yang^1,^*

***^1^*** *Department of Physiology, Meharry Medical College, Nashville, Tennessee, United States of America*

***^2^*** *Department of Pathology, Central Hospital of Wuhan, Wuhan City, People’s Republic of China*

***^3^*** *Wuhan University School of Basic Medical Science, Wuhan City, People’s Republic of China*

|  | | ITEM | RECOMMENDATION | Section/ Paragraph |
| --- | --- | --- | --- | --- |
| 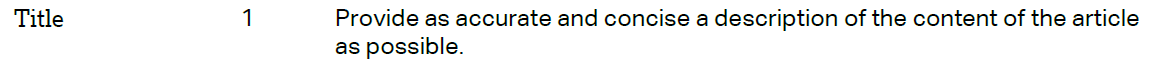 | | | Title |  |
| 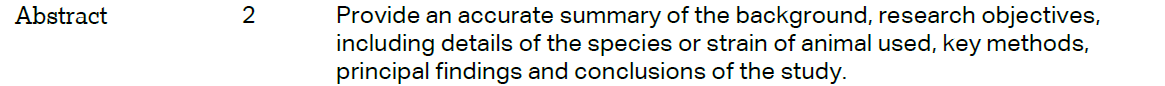 | | | Abstract  This study used a cell line.  Lipoproteins isolated from mice were used as a reagent to treat cells. |  |
| INTRODUCTION | | |  |  |
| 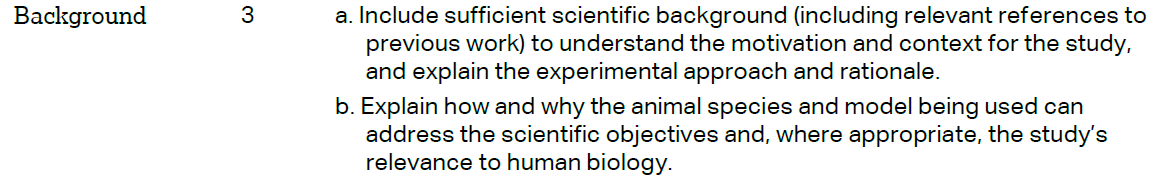 | | | paragraph 1-2  N/A  Mice were used for isolation of lipoproteins. |  |
| 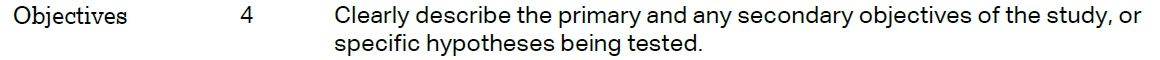 | | | Paragraph 3 |  |
| METHODS | | |  |  |
| 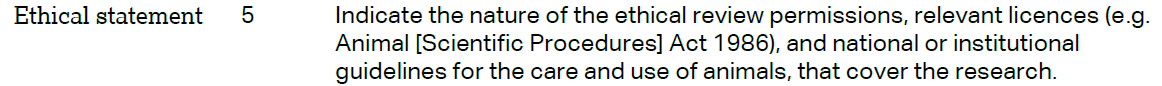 | | | Methods  Paragraph 1 |  |
| 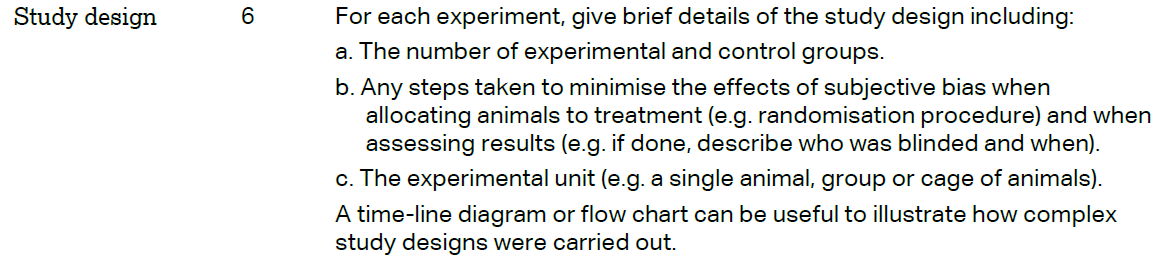 | | | Paragraph 8  Mice were used for isolation of lipoproteins. |  |
| 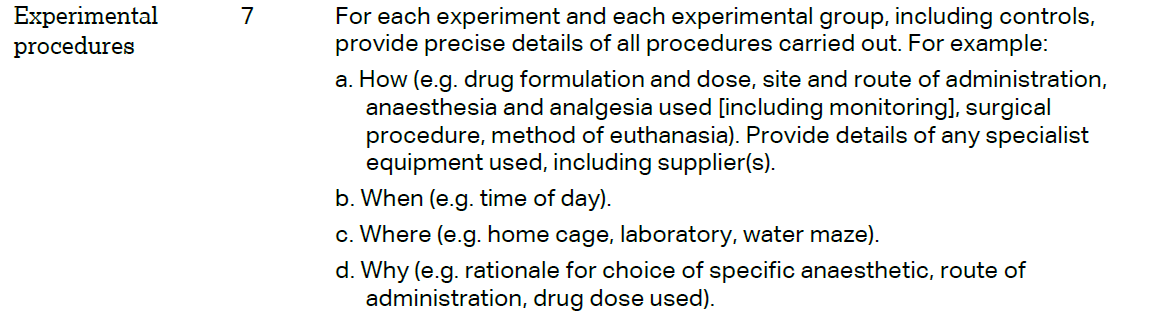 | | | Paragraph 8 |  |
| 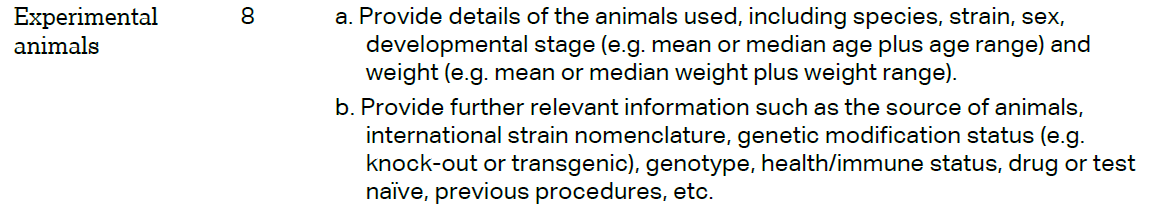 | | | Paragraph 8 |  |

| 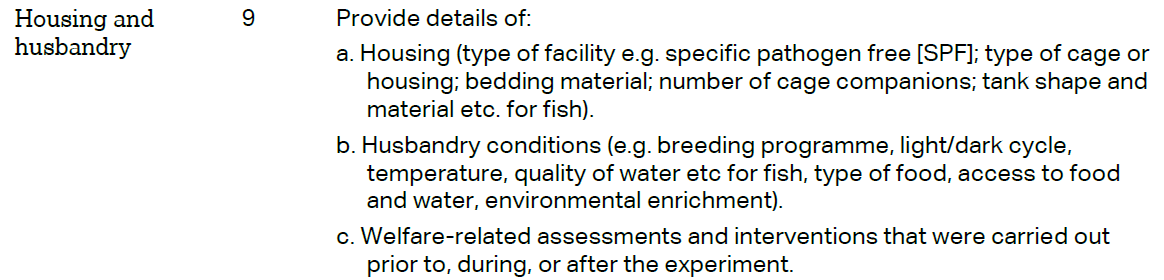 | Paragraph 8 | |
| --- | --- | --- |
| 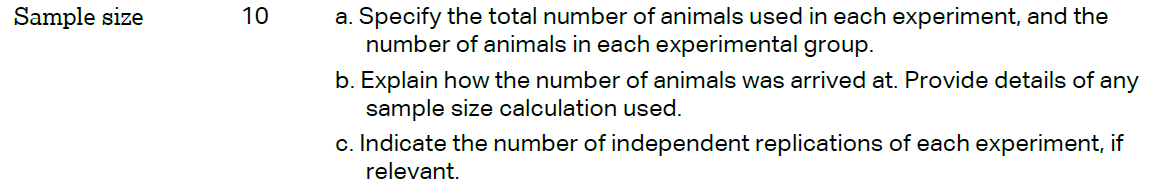 | Paragraph 8 | |
| 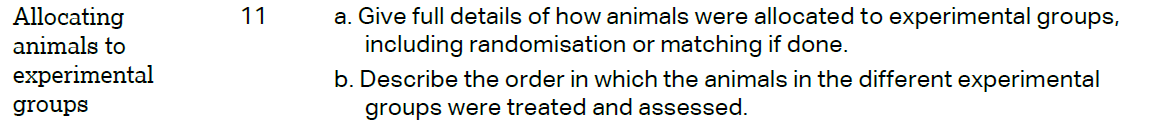 | Paragraph 8.  Mice were used for isolation of lipoproteins | |
| 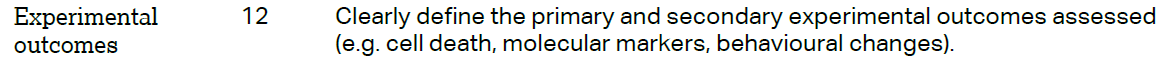 | Results  Paragraph 1-12 | |
| 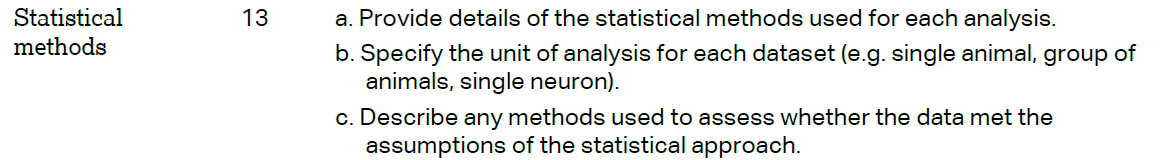 | Paragraph 14 | |
| RESULTS |  | |
| 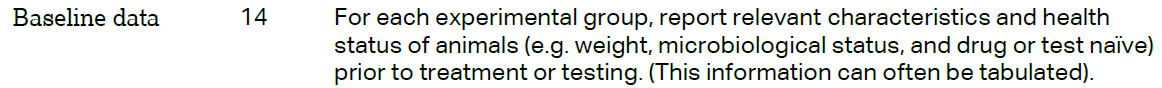 | Method  Paragraph 8 | |
| 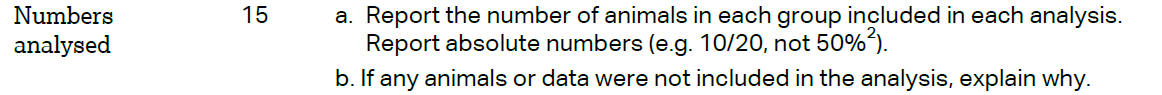 | Method  Paragraph 8  Mice were used for isolation of lipoproteins | |
| 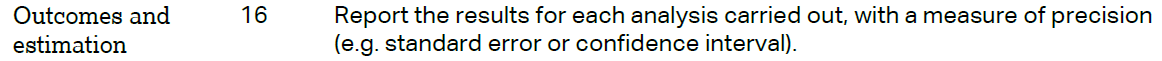 | Paragraph 1-12 | |
| 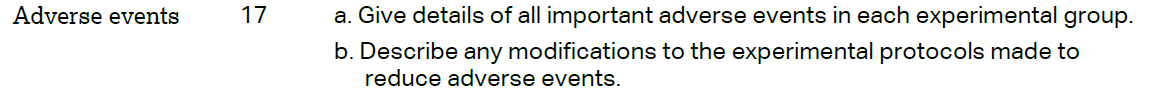 | N/A.  Mice were used for isolation of lipoproteins | |
| DISCUSSION |  | |
| 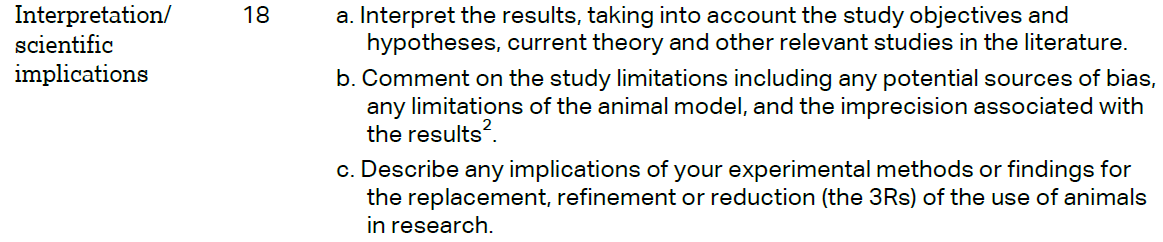 | Paragraph 1-5  N/A. this study used a cell line. | |
| 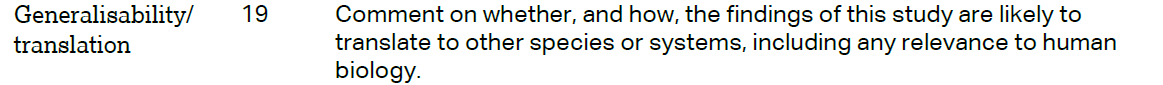 | Paragraph 6 | |
| 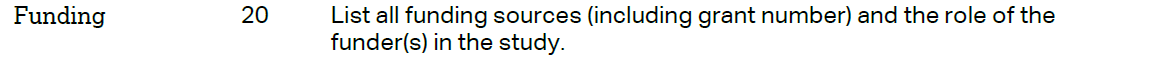 | | References  Paragraph 3 |


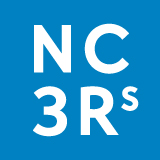

Supplement: S1 ARRIVE Checklist — (DOCX) [file pone.0136895.s001.docx]
